# The Use of the SpO_2_ to FiO_2_ Ratio to Individualize the Hypoxic Dose in Sport Science, Exercise, and Health Settings

**DOI:** 10.3389/fphys.2020.570472

**Published:** 2020-11-20

**Authors:** Jacky Soo, Olivier Girard, Mohammed Ihsan, Timothy Fairchild

**Affiliations:** ^1^Murdoch Applied Sports Science Laboratory, Discipline of Exercise Science, Murdoch University, Perth, WA, Australia; ^2^School of Human Sciences, Exercise and Sport Science, The University of Western Australia, Perth, WA, Australia; ^3^Research and Scientific Support, Aspetar Orthopaedic and Sports Medicine Hospital, Doha, Qatar; ^4^Human Potential and Translational Research Program, Yong Loo Lin School of Medicine, National University of Singapore, Singapore, Singapore; ^5^The Centre for Healthy Ageing, Health Futures Institute, Murdoch University, Perth, WA, Australia

**Keywords:** hypoxia, hypoxemia, simulated altitude, hypoxic training, oxygen saturation

## Background

Human responses to hypoxia (i.e., reduced O_2_ supply) range from immediate adjustments (minutes to hours) to prolonged adaptations (several weeks) within various physiological regulatory systems (Guillemin and Krasnow, 1997). Over the last 50 years, numerous altitude/hypoxic training modalities have been developed to capitalize on these hypoxic responses, with a view to improve athletic performance. Today, the use of hypoxia extends to therapeutic interventions (also known as “hypoxic conditioning”) (Millet et al., 2016b), an application dating back to the former Soviet union era (Serebrovskaya, 2002).

Traditional forms of altitude training include live high-train high, live high-train low, and live low-train high (LLTH) (Wilber, 2007). With the widespread availability of hypoxic chambers and portable hypoxicators, the LLTH paradigm has gained significant popularity over the last decade. This model involves exposure to hypoxia at rest or combined with exercise, while residing near sea level (Wilber, 2007; Girard et al., 2020). Altitude simulation (normobaric hypoxia) with the LLTH method is typically achieved by reducing the inspired oxygen fraction (FiO_2_), while atmospheric pressure remains unchanged. An example in professional sport, is repeated-sprint training sessions with multiple athletes, conducted at a fixed FiO_2_ of 0.145 to simulate an altitude equivalent to 3,000 m (Faiss et al., 2013).

Responses to hypoxia vary in magnitude between individuals (Friedmann et al., 2005; Chapman et al., 2011). For example, Friedmann et al. (2005) showed in 16 elite junior swimmers that the increase in erythropoietin concentration after 4 h in normobaric hypoxia (FiO_2_ 0.15) averaged ~58%, but remarkably ranged from 10 to 185%. In this regard, alternative approaches to implementing hypoxia have been proposed (Bassovitch and Serebrovskaya, 2009; Mira et al., 2020). For instance, the “arterial oxygen saturation (SpO_2_) clamp” approach (Mira et al., 2020), whereby SpO_2_ is clamped to a target/range by altering the FiO_2_ presented to each individual has been proposed as a step toward reducing variability in the responses to hypoxia.

This paper first discusses the inter-individual variability in response to hypoxic stress when using “fixed FiO_2_” as a marker of “dose,” and then examines the “SpO_2_ clamp” as an alternate approach. We then consider the usefulness of a clinical index that integrates both the external (FiO_2_) and internal (SpO_2_) stimuli to characterize individual responses to hypoxia (Rice et al., 2007), and propose its application in exercise and sport science settings.

## Defining the Hypoxic “Dose”

The fundamental variables that define the hypoxic “dose” include the severity, duration, frequency, type (normobaric or hypobaric hypoxia) and pattern of hypoxic presentation (Wilber et al., 2007; Navarrete-Opazo and Mitchell, 2014). An optimal “dose” should maximize chronic physiological benefits, whilst minimizing potential harmful consequences (e.g., headaches, dizziness). Currently, there are limited quantitative means to describe the optimal hypoxic “dose” required for planned physiological responses. Further, there is an incomplete understanding of the link between the immediate and chronic responses to hypoxia.

The environmental stress (e.g., elevation) has often been used as a predictor of the total physiological stress imposed on an individual. For example, Garvican-Lewis et al. (2016) introduced the metric termed “kilometer hours” to quantify the overall “external stress” during altitude sojourns based on the terrestrial/simulated altitude level and duration of exposure. One critique is that external load metrics does not consider the physiological stress or internal load imposed on an individual. In response, the “saturation hours” metric was suggested as a measure reflecting internal load (i.e., SpO_2_) which considers the duration at which a particular SpO_2_ is sustained during hypoxic exposure (Millet et al., 2016a).

## Nature of the Problem

A typical LLTH hypoxic training session entails a group of athletes exercising at a simulated altitude of 2,500–3,500 m (through manipulation of FiO_2_). Whilst it is tenable to expect that reduced ambient oxygen availability should decrease *in vivo* oxygenation, regulatory responses to hypoxia (e.g., increased ventilation) can influence events along the oxygen cascade to attenuate the decline in SpO_2_ (Richardson et al., 2006). Reductions in SpO_2_ at a fixed FiO_2_ vary widely due to differences in hypoxic chemosensitivity, pulmonary ventilatory limitation, hypoxic ventilatory response, arterial-venous shunting, ventilatory perfusion mismatch, and/or diffusion limitation (Weil, 2003; Chapman, 2013). Furthermore, determining an ideal hypoxic severity based on FiO_2_ *per se* is challenging since the hypoxic range falls on the steep portion of the oxyhemoglobin curve (Chapman, 2013). In other words, a small decline in partial pressure of oxygen (PO_2_) would result in a disproportionate SpO_2_ decrease. Remarkably, the variability in SpO_2_ response becomes more pronounced with increasing hypoxia severity. For instance, the SpO_2_ response of 15 healthy individuals decreased from 95–98% to 74–95% when FiO_2_ was lowered from 0.21 to 0.12 (Albert and Swenson, 2014). The heterogeneity in response to a given FiO_2_ may also result in disparity in exercise performance. For example, at an altitude of 2,100 m, elite athletes who demonstrated greater reductions in SpO_2_ also experienced larger declines in performance compared with athletes with smaller SpO_2_ fluctuations (Chapman et al., 2011). Collectively, the variability in SpO_2_ response at a given FiO_2_ suggests that some individuals may attain the planned hypoxia-induced response (i.e., those close to the average), whereas others may receive a stimulus either “too small” or “too large.” From a training perspective, a stimulus that is “too large” may inadvertently diminish beneficial gains (i.e., catabolic effect of hypoxia) from exercise training (Etheridge et al., 2011). Further, this variability in hypoxic response is reported within relatively homogenous groups (i.e., *healthy and trained*). It stands to reason that greater variability in hypoxic responses would be expected in clinical cohorts. This includes type 2 diabetes mellitus and chronic pulmonary obstructive disease, where varying degrees of mitochondrial dysfunction (Lowell and Shulman, 2005; Sangwung et al., 2020) and hypoxic ventilatory response (Weil, 2003) are evident, respectively. Considering the adoption of hypoxia training in clinical cohorts (Verges et al., 2015) along with the established variability in SpO_2_ responses to hypoxia in non-clinical cohorts, the use of FiO_2_ as a marker of “dose” requires reconsideration.

## Hypoxia Exposure—Toward An Individualized Approach

Support for the use of SpO_2_ in setting the hypoxic “dose” comes from research demonstrating that many hypoxia-induced outcomes (e.g., angiogenesis, neuromuscular adaptations) are ultimately governed by downstream events of the oxygen cascade (Ameln et al., 2005; Manimmanakorn et al., 2013). Consequently, these physiological outcomes occur in response to decreased arterial oxygen saturation, measured using SpO_2_, rather than FiO_2_ *per se* (Manimmanakorn et al., 2013). Indeed, elevated skeletal muscle adaptations (e.g., transcript expression of mitochondria biogenesis) to hypoxic training are proportional to the magnitude of SpO_2_ decrease (Schmutz et al., 2010). Methods of clamping SpO_2_ include prior oxygen titration to predetermine the optimal FiO_2_ (McKeown et al., 2019) and manual (Mira et al., 2020) or automatic adjustments (Ng et al., 2016; Bayer et al., 2017) (requiring a biofeedback mode) during the actual session. A possible concern of the “SpO_2_ clamp” approach—particularly when oxygen delivery is manually adjusted—is the accuracy of SpO_2_ responses. This is because SpO_2_ does not decrease proportionally with FiO_2_, due to the sigmoidal relationship between PO_2_ and SpO_2_. That said, studies which have attempted to clamp SpO_2_ to a specific target, or within a 3–10% range, report standard deviation values of <5% during both passive (Törpel et al., 2019) and active (Mira et al., 2020; Törpel et al., 2020) hypoxic exposure.

## SpO_2_ to FiO_2_ Index

Oxygen therapy is routinely prescribed for patients with lung conditions (e.g., in severe COVID-19 cases) experiencing hypoxemia (Alhazzani et al., 2020). To mitigate risks associated with hypoxemia and hyperoxia-related lung injury, oxygen delivery is individually titrated within a tight range. The calculation of the pulmonary shunt fraction is the preferred clinical assessment of the oxygenating capacity of the lungs, although arterial oxygen partial pressure (PaO_2_) and SpO_2_ have been proposed as surrogate measurements of oxygenation (Zetterstrom, 1988). In order to assess the severity of hypoxemia in ventilated patients (where supplemental oxygen is used to maintain SpO_2_ within a normal/safe range) the PaO_2_ to FiO_2_ ratio, and later the SpO_2_ to FiO_2_ ratio (SF), were proposed (Horovitz et al., 1974; Rice et al., 2007). To illustrate, a healthy individual at sea level with a SpO_2_ of 98% would have a SF value of 467 (i.e., 98/0.21). Lower SF values are indicative of reduced oxygenating capacity, and is used, for instance, to diagnose patients with acute respiratory distress syndrome (SF values ≤ 235) and acute lung injury (SF values ≤ 315) (Rice et al., 2007). Unlike previous approaches, the SF ratio considers both the internal and external stimuli which allows for comparison between individuals/groups. Furthermore, the SF index is readily accessible and easy to interpret, which therefore represents an appealing tool for the early assessment of patients with potential respiratory disorders.

## Future Directions

Moving beyond the conventional “fixed FiO_2_” approach, an individualized approach to administering hypoxia may consist of a combination of strategies such as (1) a prior hypoxia test to elucidate variability in responsiveness to hypoxia, (2) altering severity of hypoxia individually to regulate SpO_2_ within a tightly defined range, and (3) reporting the inter-individual variability based on the SF index.

**Figure 1:**
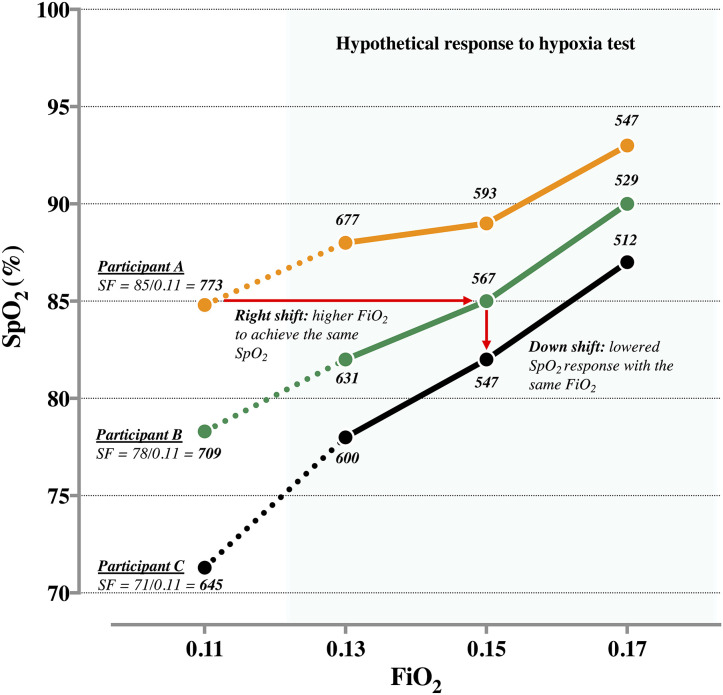
Individual arterial oxygen saturation (SpO_2_) response of participants A, B, and C at fractional inspired oxygen (FiO_2_) of ~0.11. Hypothetical SpO_2_ response to a hypoxia test at FiO_2_ of 0.13, 0.15, and 0.17. Corresponding SpO_2_ to FiO_2_ ratio (SF) are presented at each data point.

A hypoxia test can be used to estimate the trajectory of SpO_2_ to hypoxia, and in turn, inform decisions on the hypoxic “dose.” Figure 1 depicts the hypothetical SpO_2_ responses of participants A, B, and C during a decremental titration using FiO_2_ of 0.17, 0.15, and 0.13. As illustrated, the corresponding responses form an abbreviated individual-specific oxyhemoglobin curve. In this example, with a lower SpO_2_ response to a given FiO_2_, participant C displays the highest response to hypoxia compared to participants A and B; this is represented by rightward and downward shifts of the abbreviated oxyhemoglobin curve. Participant C would likely require a higher FiO_2_ (i.e., milder hypoxia) to record similar SpO_2_ values as participants A and B. For instance, if the target SpO_2_ is 85%, the approximate FiO_2_ for participants A, B, and C would be 0.11 (SF: 85/0.11 = 773), 0.15 (85/0.15 = 567), and 0.16 (85/0.16 = 531), respectively. The corresponding SF values may then provide clarity on the inter-individual variability in response to hypoxia, wherein a low SF value indicates a high sensitivity to hypoxia.

Where a “fixed FiO_2_” approach is used to administer hypoxia, the SF index may also provide similar information about inter-individual variability. At a FiO_2_ of ~0.11, for instance, the SpO_2_ response of participants A, B, and C are 85, 78, and 71%, equating to SF values of 773, 709, and 645, respectively (Figure 1). By establishing threshold values for SF, distinct groups can be identified and clustered for training purposes, to increase likelihood of achieving similar physiological responses.

## Challenges for Implementation

The appeal of the “fixed FiO_2_” approach, is the ease of implementation, for example, in an environmental chamber where a group of athletes can train together. Comparatively, whilst an individualized approach may produce a more consistent hypoxic response, such an approach would likely require personalized equipment and/or prior preparations (e.g., titration of “dose”). That said, an individualized approach to administering hypoxia would be applicable across the spectrum from clinical cohorts to elite-level athletes. However, it should be highlighted that the SF index is a measurement that does not consider the type of hypoxia exposure (i.e., hypobaric vs. normobaric). Since greater desaturation is associated with hypobaric than normobaric hypoxia for a matched inspired PO_2_ (Saugy et al., 2014), SF values may not be strictly equivalent between terrestrial and simulated hypoxia, and therefore should not be used interchangeably.

## Conclusion

Traditionally, hypoxic training has adopted a universal approach, wherein all individuals receive the same absolute hypoxia stress (i.e., FiO_2_). Whilst highly practical, substantial inter-individual variability in response to a given FiO_2_ is indisputable. The implication being, that some individuals attain the appropriate hypoxia-related adaptations, whereas others may receive potentially harmful or ineffective stimuli. Similar to the individual tailoring of training variables, we suggest that the administration of hypoxia requires an individualized approach. We therefore propose that the SF index (i.e., SpO_2_ to FiO_2_ ratio)—which is already widely adopted in clinical settings—can also be used by exercise physiologists and sport scientists to gauge an individual's response to hypoxia. This may ultimately offer a more pragmatic approach toward defining physiologically distinct groups of individuals and enable a tailored level of FiO_2_.

## Author Contributions

All authors listed have made a substantial, direct and intellectual contribution to the work, and approved it for publication.

## Conflict of Interest

The authors declare that the research was conducted in the absence of any commercial or financial relationships that could be construed as a potential conflict of interest. The handling editor declared a past co-authorship with the authors JS and OG.
